# Nutrition knowledge, weight loss practices, and supplement use in senior competition climbers

**DOI:** 10.3389/fnut.2023.1277623

**Published:** 2024-01-17

**Authors:** Edward Gibson-Smith, Ryan Storey, Marisa Michael, Mayur Ranchordas

**Affiliations:** ^1^Academy of Sport and Physical Activity, College of Health, Wellbeing and Lifestyle, Sheffield Hallam University, Sheffield, United Kingdom; ^2^Sport Industry Research Centre, College of Health, Wellbeing and Lifestyle, Sheffield Hallam University, Sheffield, United Kingdom; ^3^Real Nutrition LLC, West Linn, OR, United States

**Keywords:** climbing, nutrition, bouldering, sport climbing, weight loss, competition, supplements

## Abstract

**Introduction:**

Sport climbing has gained increased scientific attention, including studies investigating the dietary habits and nutritional requirements of climbers; however, significant gaps in the literature remain. An assessment of nutritional knowledge, weight loss for competition, and supplement use has not been previously reported in senior competition climbing athletes.

**Methods:**

Fifty climbers (26 male, 24 female; BMI 21.6 ± 1.9; 23.7 ± 5.2 years) participated in the study. Participants answered a 72-item questionnaire, comprised of demographic data and three main sections to assess general and sports nutrition knowledge, weight loss strategies, and supplement use.

**Results:**

The mean nutrition knowledge score was ‘average’, with considerable individual variation (53.5 ± 11.1 %). There were no significant sex differences in the general (GNK) or sport (SNK) nutrition knowledge scores, or effect of age. Significantly higher knowledge was demonstrated by national vs. international athletes for the GNK scores (11.09 ± 1.58 vs. 9.58 ± 1.75; *p* = 0.028). Participants scored well in questions concerning protein, carbohydrates, alcohol, and supplements, and conversely, performed poorly in hydration and micronutrient related questions. Less than one-fifth of respondents had access to a dietitian. Forty-six percent of males and 38% of female climbers reported intentional weight loss for competition on at least one occasion. Of those, ~76% reported utilizing concerning practices, including methods that conform with disordered eating and/or eating disorders, dehydration, vomiting, and misuse of laxatives. Approximately 65% of athletes reported using at least one nutritional supplement in the previous 6 months, with 44% reporting multiple supplement use. There was no significant difference in supplement use between sexes or competition level.

**Discussion:**

Due to the established importance of nutritional intake on athlete health and performance, educational support should be employed to improve knowledge in climbers and address shortcomings. Moreover, intentional weight loss for climbing competition is common, with most athletes achieving ~3–8% body weight loss over ≥2 weeks. It is crucial that professionals working with competitive climbers are vigilant in identifying athletes at risk of concerning weight management and establish referral pathways to the appropriate specialist services. High quality intervention trials to assess the efficacy of ergogenic aids in climbing remains inadequate.

## 1 Introduction

Climbing as a competitive sport resembling the modern format started in 1985 in Bardonecchia, Italy, and the first competition event on an artificial climbing wall was held the following year (1). Encompassing climbing disciplines of lead, bouldering, and speed, sport climbing has grown to immense popularity since, with over 25 million climbers in around 150 countries worldwide (1). Sport climbing made its debut at the Olympic Games at Tokyo 2020 following previously successful inclusion at the youth games and has been selected to be one of the four new sports of Paris 2024, alongside breaking, surfing, and skateboarding (1).

The most recognized climbing competitions are governed by the International Federation of Sport Climbing (IFSC) and divided into 3 sub-disciplines of indoor climbing; speed, bouldering, and lead. The 2020 Olympic Games event was the first to require senior athletes to compete in all three sub-disciplines, with one overall winner chosen from each sex, however, this has been modified for the Paris 2024 programme, where speed climbing has been allocated an individual medal and will take place as a separate event (1).

Lead climbing takes place on artificial walls which must permit a route of at least 15 m in length and 3 m in width. Climbers must carry their rope as they ascend, clipping into carabiners placed at fixed protection points in the route. Scoring is determined by the furthest hold used in a controlled manner, effectively representing how high up the route the athlete gets before they fail. Attempts in which multiple athletes complete the route or reach the same hold are differentiated using the time taken to complete their attempt. In IFSC competitions, qualification requires athletes to climb on two non-identical routes separated by at least 50 min rest. In scenarios where more than one round takes place in a day, these must be separated by at least 2 h. Lead climbing attempts are notably longer than in other disciplines, lasting from 2 to 7 min with a larger proportion (~38%) spent in static positions (2). To date, few studies have investigated the energy cost and profile of lead climbing, however, the contributions of the aerobic, anaerobic lactic, and anaerobic alactic systems during difficult climbing have been reported as 41.9, 22.3, and 35.8%, respectively (3).

Bouldering is performed without the use of ropes on lower walls with crash mats used for protection. Routes must be designed so that the lower body of the climber should not exceed 3 m above the protection. Athletes are allowed multiple attempts at a route or “problem” within a 5-min period. IFSC competitions require athletes to attempt 5 routes in the qualification round, followed by 4 routes in the semi-final and final rounds. Scoring is determined by the number of problems an athlete completes in each round, alongside the number of attempts made. Athletes typically attempt a problem 3 times, with attempts lasting ~30–40 s, and ~ 115 s of recovery in between attempt (4). Ascents generally feature low static periods (~25%) and an exercise-to-recovery ratio in the forearm flexors of ~13:1 (4). This could be due to the steeper routes typically used in bouldering combined with the precarious nature of the hand and foot holds which enhance the difficulty of routes, allowing fewer opportunities find stable positions to reduce force through the upper limbs. As the energy release from PCr hydrolysis is significantly reduced after ~10 s of high-intensity exercise (5), it could be assumed that bouldering athletes will rely on larger contributions of the lactate energy systems for the provision of energy; indeed, La Torre et al. (6) reported mean blood lactate levels following national competitions of 6.2–6.9 mmol·L^−1^.

Speed climbing requires a pair of athletes to race side-by-side to the top of two identical routes set on a 15 m artificial wall. Competitors will make one attempt in each lane separated by a minimum of 5 min, with the winner decided by the lowest aggregate time over the two runs. Since 2007, the IFSC have used a standardized wall and hold configuration for all competitions and world record attempts. IFSC certified speed climbing walls are slightly overhanging (not more than 5 degrees) and feature a route with a difficulty which would be achievable by most recreational climbers. The timing of attempts begins after a countdown of beeps, and the clock is stopped by the athlete striking a mechanical–electrical pad at the top of the route. Athletes are protected from falls using a rope attached at the top of the wall to an auto-belay system. Speed climbing events feature an all-out sprint of powerful and dynamic moves, with elite efforts typically lasting 5–6 s in duration, emplacing the anaerobic demand (7).

Based on the significant discrepancies in the physiological demands of each discipline, it is reasonable to assume a variance in the nutritional requirements and practices of the athletes who specialize in each event (8, 9). In line with growing public interest and participation, sport climbing has gained increased scientific attention, including studies investigating the dietary habits and nutritional requirements of climbers (8, 10–12). Nevertheless, significant gaps in the literature remain, and little is known regarding the existing nutritional knowledge, strategies of weight loss, or supplement use in competition climbers, warranting further research.

The positive influence of an individualized dietary intake on sports performance and recovery is well established and evidenced by the publication of internationally recognized expert consensus statements outlining guidelines for the optimal intake and timing of food, fluid, and supplements (13–15). Despite this, research shows that many athletes, including climbers, have sub-optimal dietary intakes (8, 10, 16); time constraints, financial considerations, limited cooking skills, and access to cooking facilities have been proposed as potential barriers to achieving optimal dietary intakes in athletes (17). Furthermore, cultural background, taste preferences, appetite, attitude toward nutrition, nutrition knowledge, and access to professional support have been highlighted in a review of factors influencing athletes’ food choices (18).

A 2011 systematic review of the nutrition knowledge of recreational and elite athletes, reported a positive correlation between general and sport specific nutrition knowledge, and good quality dietary intake (19). Although this relationship appears to be weak to moderate (16, 19), nutrition knowledge is regarded as one of the few modifiable determinants of dietary behaviors (20). Thus, educational interventions centered on addressing gaps in athlete knowledge and adherence to expert guidelines remains a common approach in the support of sports dietitians (18).

Weight loss methods have been anecdotally practiced by climbers for decades, with athletes generally believing that by reducing their body mass they can improve their strength to weight ratio, reduce load on the extremities, and subsequently improve performance. However, there is a lack of published research to support this notion, with most studies finding little impact of body weight on performance among high-level athletes (21, 22). Although research regarding weight loss practices of climbers is scarce, early indications suggest that climbers face similar challenges to athletes in other weight-sensitive sports, which include increased risk of iron deficiency, chronic energy deficiency, and disordered eating (10, 23).

Dietary supplements are used at all levels of sports performance and a prevalence of 40–100% has been reported in athletes depending on the sport, level of competition, method of data collection, and definition of supplements used (24). Gibson-Smith et al. (10) investigated the prevalence of supplement use in climbers, finding that 45% of athletes took supplements, with protein powder, vitamin tablets, and fish oil capsules being the most prevalent, similar to the findings of Sas-Nowosielski and Judyta (25). Peoples et al. (26) found that protein, caffeine, and energy bars were the most commonly used supplements among climbers, with elite climbers being more likely than their intermediate counterparts to use protein and caffeine supplements.

There have been numerous supplement intervention trials in climbers over the last decade to quantify the effect of caffeine, creatine, beta-alanine, and other supplements on climbing performance. However, there is a lack of consistency in methods of measuring climbing “performance”, with the vast majority of studies relying on methods with unproven reliability or lacking ecological validity. More specifically, methods of forearm muscle oxygenation (27), campus board exercises (28), arm-crank Wingate tests (29), pull-up repetitions and/or velocity (30), hand grip strength (31), and time to climb boulders and sport routes (32) have all been used in supplement intervention trials, providing little comparative data or indication of which methods are reflective of real-world climbing performance, and which supplements provide the most meaningful change.

An assessment of nutritional knowledge or strategies of weight loss has not been previously reported in climbing athletes. Furthermore, only one previous study has assessed prevalence or sources of influence of supplement use in recreational climbers (33), and none have examined competition climbers specifically. Therefore, the aim of this study was to firstly, assess the current nutrition knowledge of competition climbers and identify gaps which may inform education programs to support health and performance; secondly, develop a greater understanding of the magnitude, sources of influence, and strategies of weight loss in competition climbers; and lastly, to identify the prevalence, rationale, and sources of influence of supplement use in competition athletes.

## 2 Methods

### 2.1 Participants

Participants were primarily recruited from the semi-finals stage of two international climbing competitions (CWIF 2019/20, Sheffield, United Kingdom), the 2019 British Bouldering Championship (Sheffield, United Kingdom), and the British Universities & Colleges Sport (BUCS) finals (Sheffield, United Kingdom). An electronic version of the questionnaire was also created and advertised on social media and online climbing forums. Fifty climbers (24 females, 26 males) volunteered to participate. Participants were required to meet the following inclusion criteria: age ≥ 18 years, competing at university/collegiate, national, or international level within the previous 12 months, and actively training for competition. Ethical approval was received from Sheffield Hallam University Research Ethics Committee in January 2019 (ER10121205). Following written study briefings, participants provided written or digital informed consent to participate, and for the data collected to be used freely for publication.

### 2.2 Questionnaire

Participants answered a 72-item questionnaire, in paper or electronic format. The electronic format was designed and hosted using a bespoke online survey platform developed by the Sports Industry Research Centre (SIRC) at Sheffield Hallam university. The questionnaire was comprised of three main sections to assess sport nutrition knowledge, weight loss strategies, and supplement use. The questions for each section were derived from three pre-validated questionnaires used in previous studies investigating the respective themes of each main section (34–36). Participant demographic data (e.g., sex, nationality, competition level, primary discipline) were also gathered. The questionnaire took approximately 20–40 min to complete and outlined within the participant information sheet. Questions not relevant to an individual participant could be omitted (e.g., if a participant did not partake in intentional weight loss *and/or* consume dietary supplements).

#### 2.2.1 General and sports nutrition knowledge

The Abridged Nutrition for Sport Knowledge Questionnaire (A-NSKQ) is a brief and reliable tool designed and validated by Trakman et al. (36, 37) to assess general nutrition knowledge (GNK) and sports nutrition knowledge (SNK). The A-NSKQ has 37 items (GNK = 17; SNK = 20) and covers the same key topics assessed in the 89-item NSKQ (weight management, macronutrients, micronutrients, supplementation, sport nutrition, and alcohol). However, typical completion time of the A-NSKQ is around half the time required to complete the NSKQ (12 vs. 25 min) with comparable reliability and validity (36), and therefore, deemed more appropriate for use when administered concurrently with additional tools.

#### 2.2.2 Weight loss strategies

A questionnaire used to assess weight loss strategies and concerning dietary habits in mixed martial arts [(34); originally developed and validated by (38)] was modified appropriately for use with climbing athletes. Modifications included adapting terminology to suit the sport of climbing (e.g., disciplines of climbing rather than combat sports) or with the use of generalized language (e.g., weight loss directly before a “competition”, rather than “weigh-in”). This section of the questionnaire contained 11 questions including typical pre-competition or competition season weight loss, influences of weight management, and weight loss behaviors.

#### 2.2.3 Nutritional supplement habits

The final section of the questionnaire contained 14 questions to assess nutritional supplement use including reasons for use/nonuse, side effects, sources of influence, and attitudes toward supplementation. These questions were derived from a previous study investigating supplement habits in an athletic population (35).

### 2.3 Statistical analysis

Statistical analysis was performed using SPSS software (version 24, IBM, United States). Data was checked for homogeneity of variance using Levene’s test, and normality using Shapiro–Wilk’s. The differences in variables between groups (e.g., competition level) were analyzed using an independent-samples one-way ANOVA test. Where non-parametric data was identified, the independent-samples Mann–Whitney U test was used. Statistical significance for all tests was set at *p* ≤ 0.05. A Spearman’s correlation coefficient determined the relationship between data sets (e.g., age and GNK). Correlation values (R) were set as <0.2: weak correlation, 0.5: medium correlation, and > 0.8: strong correlation (39). Supplement use prevalence data were evaluated by sex and competition level using chi-square analyses. Data are presented as means ± standard deviation (SD), unless otherwise stated.

## 3 Results

### 3.1 Participant demographics

Participant anthropometric data and demographics are shown in Tables 1 2. Fifty competition climbers (*n* = 26 male, *n* = 24 female) aged 18–37 (mean age 23.7 ± 5.2 years) participated in the study. The athletes reported a mean competition frequency of 7.6 ± 1.9 in the previous 12 months. Average BMI was 21.6 ± 1.9; a BMI of <18.5, defined as potentially “underweight” (40) was reported in two participants.

**Table 1:** Anthropometric data.

	Male (*n* = 26)		Female (*n* = 24)	
	Mean ± SD	Range	Mean ± SD	Range
Age (years)	24.3 ± 6.0	18.0–37.0	23.1 ± 4.3	18.0–33.0
Mass (kg)	66.9 ± 7.4	54.0–79.0	60.4 ± 7.2	45.0–78.0
Height (cm)	175.7 ± 8.0	160.0–188.0	167.6 ± 4.9	160.0–178.0
BMI	21.6 ± 1.3	18.9–24.4	21.5 ± 2.4	16.9–27.4

BMI: body mass index.

**Table 2:** Participant demographics.

	*n*	% of sample
Age started competitions		
5–9	8	16
10–14	22	44
15–19	9	18
20–24	7	14
>25	4	8
*Discipline*		
Lead	8	16
Bouldering	34	68
Combined	8	16
*Competition level*		
University/Collegiate	12	24
National	12	24
International	26	52
*Nationality*		
United Kingdom	27	54
Other European	12	24
North America	9	18
Other	2	4

### 3.2 General and sports nutrition knowledge

The mean scores and range on the A-NSKQ are reported in Table 3.

**Table 3:** Mean scores on the A-NSKQ.

	Mean score (%)	Range (%)
Total	53.5 ± 11.1	25.0–75.0
GNK	62.5 ± 12.3	37.5–87.5
SNK	46.7 ± 13.8	15.0–75.0

Mean ± standard deviation, GNK, general nutrition knowledge; SNK, Sports nutrition knowledge.

#### 3.2.1 Individual items and gaps in knowledge

The authors determined a correct response rate of ≥75% or ≤ 25% to be notable for individual items.

##### 3.2.1.1 Macronutrient knowledge

Items with a notably high correct response rate included, “protein needs can be met by a vegetarian diet without the use of supplements” (82%), “consuming carbohydrate during exercise will assist in maintaining blood glucose levels” (76%), “fat is involved in immunity” (75%), and “a banana is high in carbohydrate” (82%). Conversely, only 19% correctly identified cottage cheese as a “low-fat” food option.

##### 3.2.1.2 Micronutrient knowledge

In regard to micronutrient knowledge, 75% of respondents were able to correctly identify the statement “vitamins provide the body with energy” as false. However, very few athletes correctly answered a question relating to the role of vitamin B in the delivery of oxygen to muscles (7%) or understood an athlete’s needs for magnesium and calcium (8%).

##### 3.2.1.3 Hydration

Athlete knowledge on hydration for sports performance was poor; just 22% of the athletes identified the correct hydration strategy during exercise, with only 10% of athletes correctly identifying the reason they should drink during exercise (i.e., to maintain plasma volume).

##### 3.2.1.4 Alcohol

The highest correct response rate was seen within the alcohol category. Almost all athletes (96%) correctly identified that “alcohol contains calories that can lead to weight gain” and that “alcohol can reduce recovery from injury” (90%).

##### 3.2.1.5 Supplements

Most athletes knew that “supplement labels may contain false or misleading information” (84%), or that taking testosterone is banned in sport (88%).

#### 3.2.2 Individual characteristics and knowledge

##### 3.2.2.1 Sex differences

There were no significant differences between males and females for the GNK (10.10 ± 1.80 vs. 9.70 ± 2.00, *p* = 0.34) or SNK scores (9.40 ± 3.20 vs. 9.30 ± 2.30, *p* = 0.79).

##### 3.2.2.2 3.3.2.2 Competition level

ANOVA analysis demonstrated a significant difference in knowledge scores between competition levels (*p* = <0.05). Bonferroni post-hoc analysis revealed higher nutrition knowledge scores in national vs. international athletes, reaching significance for the GNK scores (11.09 ± 1.58 vs. 9.58 ± 1.75, *p =* 0.03), and almost reaching significance for the SNK scores (11.0 ± 2.14 vs. 8.65 ± 2.83, *p* = 0.053*).*

##### 3.2.2.3 Access to dietician/nutritionist

Less than one-fifth of respondents (18.4%, *n* = 9) had access to a dietitian. There was no significant difference in the combined scores (GNK + SNK) between groups with or without access to a dietician (20.00 ± 4.77 vs. 19.08 ± 3.90, *p =* 0.54).

##### 3.2.2.4 Age correlation

There was no significant correlation between participant age and GNK, SNK, or combined scores (*R* = 0.22, *p* = 0.12; *R* = 0.24, *p* = 0.09; *R* = 0.21, *p* = 0.15).

### 3.3 Weight loss practices

#### 3.3.1 History and magnitude of weight loss

The history and magnitude of weight loss is reported in Table 4.

**Table 4:** History and magnitude of weight loss.

		Male	*n*	Female	*n*
Have you ever lost weight for competition (% yes)		46	12	38	9
At what age did you begin to lose weight for competition? (mean ± SD)		22.4 ± 5.7		19.9 ± 4.8	
Do you attempt to lose weight on the day before or the day of competition? (~% yes)		8	1	12	1
How many times have you lost weight for competition? (~%)	1–2	58	7	12	1
	3–5	25	3	75	7
	6–10	0	0	12	1
	>10	17	2	0	0
How much weight would you typically lose? (%)	<2 kg	0	0	62	6
	2–5 kg	92	11	38	3
	6–10 kg	8	1	0	0
How much time do you lose the weight over? (%)	<48 h	0	0	12	1
	1–2 weeks	17	2	12	1
	2–4 weeks	25	3	45	4
	>1 month	58	7	34	3

#### 3.3.2 Ranking of influence on weight loss practices

**Figure 1:**
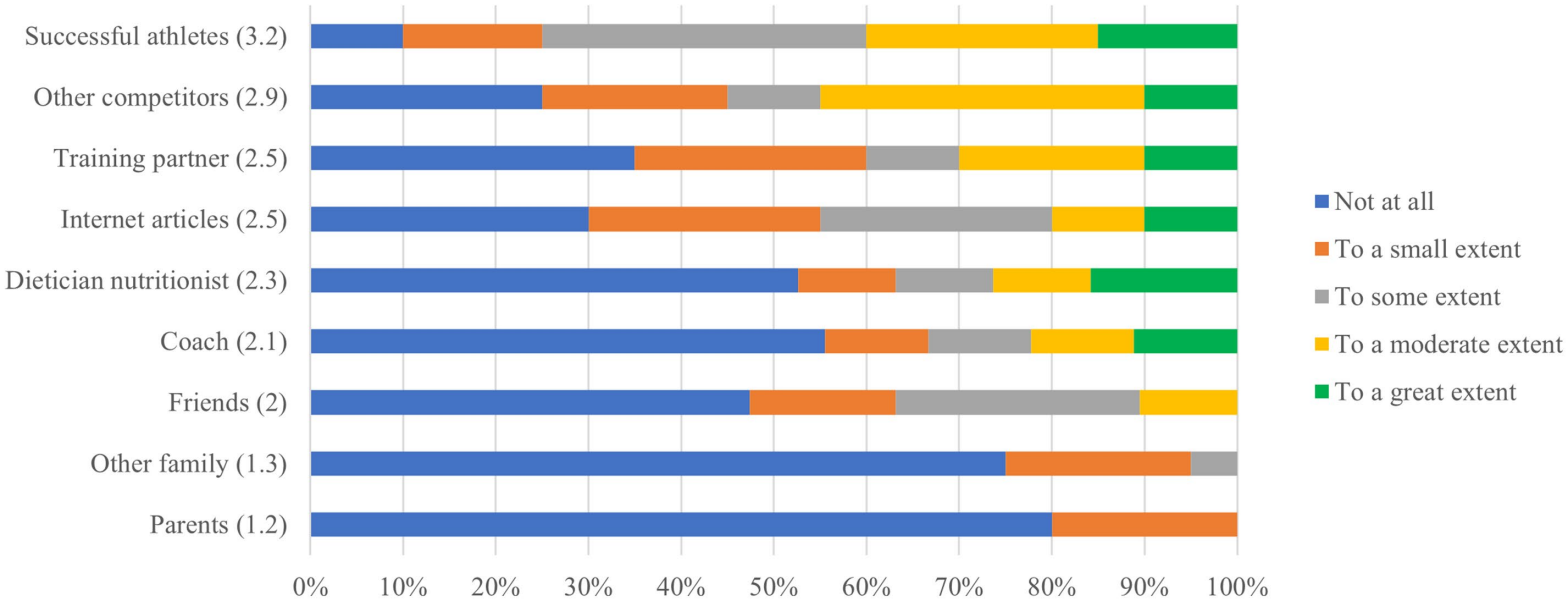
Ranking of influence on weight loss practices.

Figure 1 shows the ranking of influence on weight loss practices. The top 3 sources of influence were successful athletes, other competitors, and internet articles, with dietician/nutritionist and coach, ranking 5th and 6th, respectively.

#### 3.3.3 Ranking of prevalence of weight loss practices

**Figure 2:**
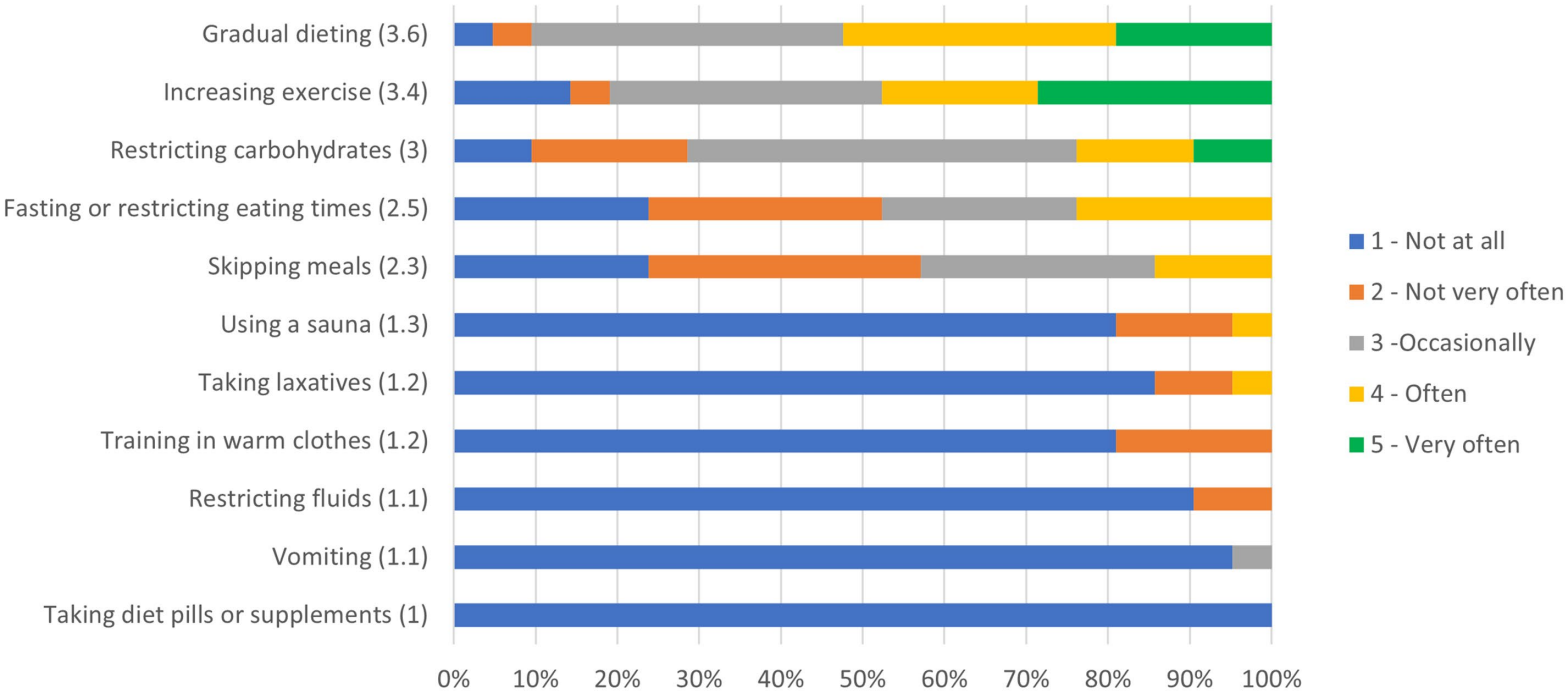
Ranking of prevalence of weight loss practices.

Figure 2 shows the ranking of prevalence of weight loss practices.

#### 3.3.4 Prevalence of concerning weight loss practices

Table 5 provides a summary of responses reporting concerning weight loss practices. 76% (16 of 21) of participants who reported intentionally losing weight for competition are practicing concerning weight loss methods, including skipping meals, using a sauna, training in warm clothes, taking laxatives, restricting fluids, or vomiting. The prevalence of concerning weight loss practices appears to be similar between males and females (~75% vs. 78%).

**Table 5:** Prevalence of concerning weight loss practices.

	*n*		
	Male	Female	Total
Skipping meals	9	7	16
Using a sauna	3	1	4
Training in warm clothes	4	0	4
Taking laxatives	3	0	3
Restricting fluids	2	0	2
Vomiting	1	0	1

### 3.4 Supplement use

#### 3.4.1 Prevalence of supplement use

**Figure 3:**
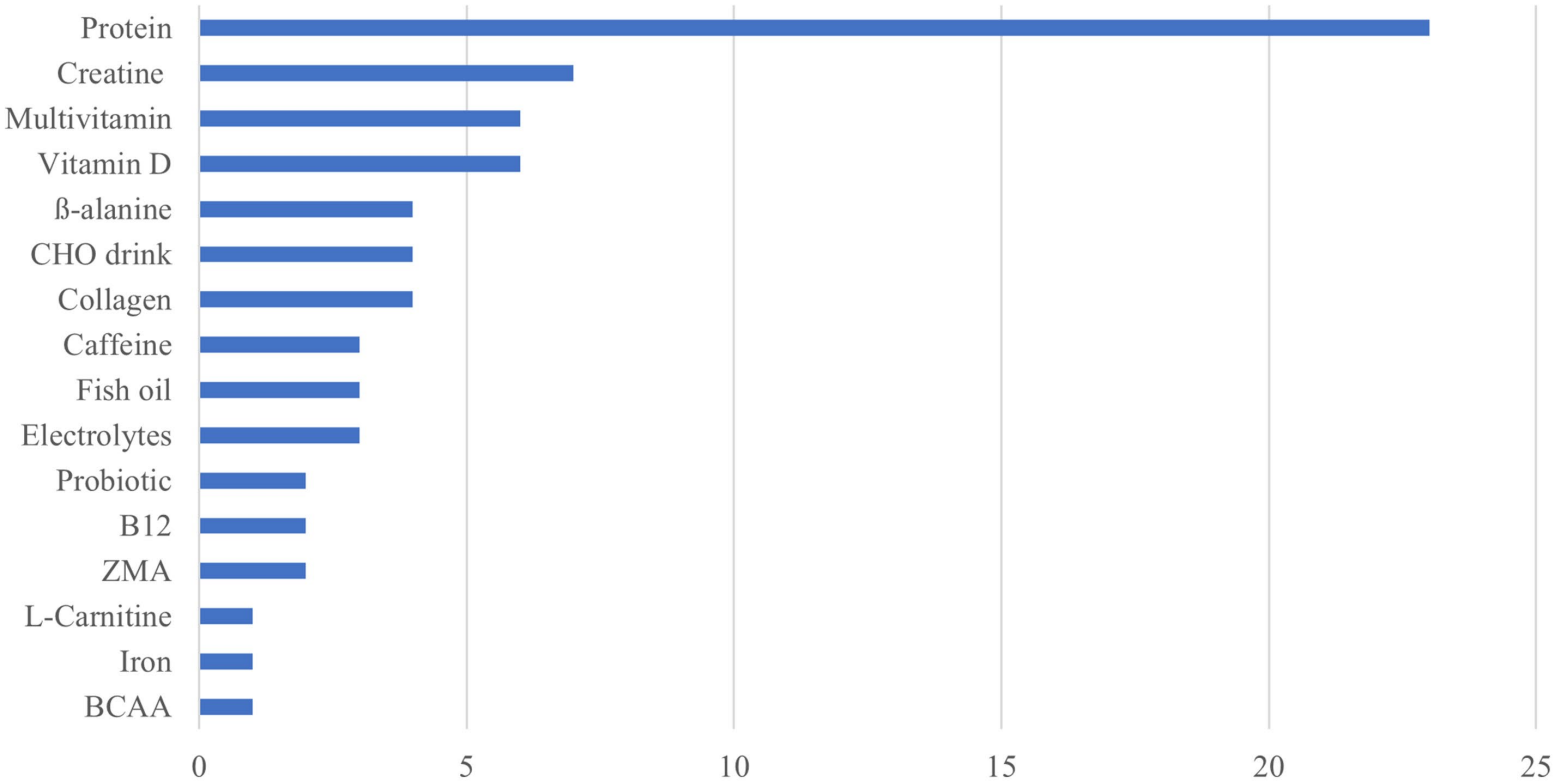
Frequency distribution for the type of nutritional supplements used.

The frequency distribution for the type of nutritional supplements used is shown in Figure 3.

Approximately 65% (*n* = 34) of athletes reported using at least one nutritional supplement in the previous 6 months, with 44% (*n* = 22) reporting multiple supplement use. Of those, ~46% of nutritional supplements (NS) were used daily (at least 4–5 times per week), ~29% were used before, during, or after training, ~ 20% used occasionally, and only ~5% were competition specific.

The three most popular supplements used exclusively for health included vitamin D (*n* = 6), multivitamins (*n* = 6), and fish oil (*n* = 3). The three most popular supplements used exclusively for performance included creatine (*n* = 7), CHO drinks (*n* = 4), and beta-alanine (*n* = 4). The three most common outlets athletes obtained supplements were the internet (36%), health food/sports shops (21%), and pharmacies (19%).

Fourteen percent of all athletes (*n* = 7) reported having experienced a negative effect from using nutritional supplements, such as gastrointestinal/digestive problems (protein, BCAA, iron, biotin) and weight gain (creatine). The top 3 reasons reported for non-use of supplements (participants could select more than one answer) were, “I do not need them” (*n* = 9), “I do not know enough about them” (*n* = 14), and “they are too expensive” (*n* = 8).

#### 3.4.2 Comparisons by sex and competition level

**Figure 4:**
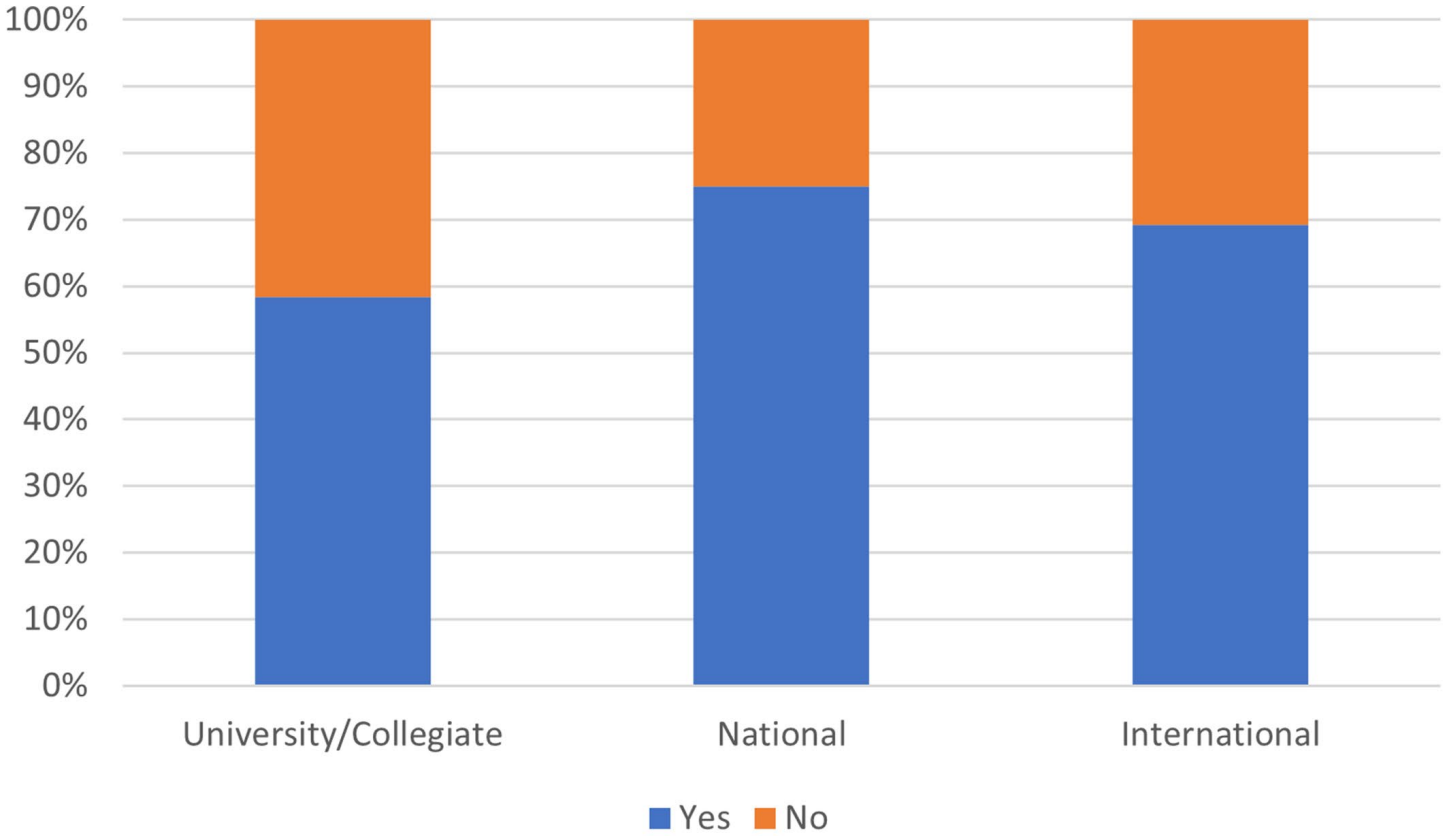
Nutritional supplement use within the competition levels in this sample.

There was no significant difference in supplement use between sexes or competition level (*p* = >0.05). Figure 4 indicates the use of NS within the competition levels in this sample.

#### 3.4.3 Ranking of influence on supplement use

**Figure 5:**
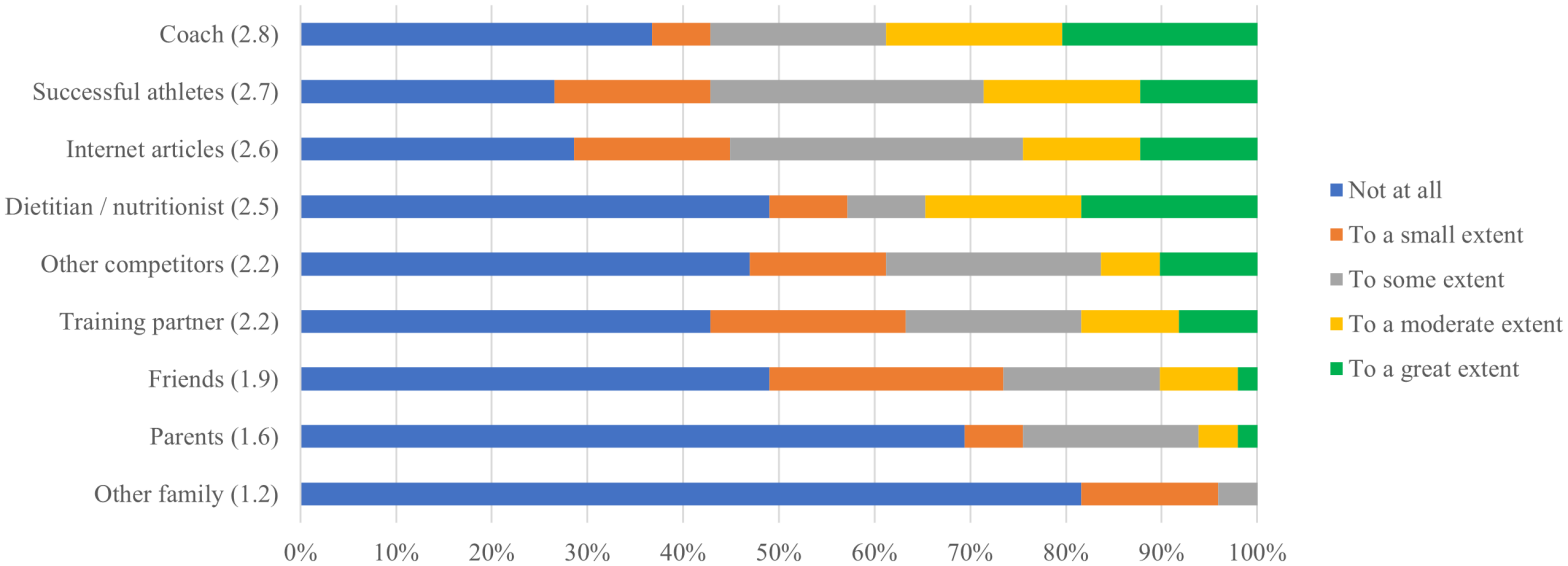
Ranking of influence of supplement use.

Figure 5 shows the ranking of influence on supplement use.

Athletes ranked their coach, successful athletes, and internet articles as their top three sources of influence. Dietitian/nutritionist ranked in 4th position, however, only ~18% of athletes reported access to a nutritionist/dietitian. Interestingly, national athletes had greater access to nutritionists/dietitians (25%, *n* = 3) compared with international (16%, *n* = 4) or collegiate level (17%, n = 2). Although, international athletes had greater access to anti-doping information (88%, *n* = 22) compared with national (8%, *n* = 1) or collegiate level (8%, *n* = 1).

#### 3.4.4 Influence of nutrition knowledge

There was no significant difference in sports nutrition knowledge (SNK) (9.76 ± 2.87 vs. 8.4 ± 2.29, *p* = 0.11) or general nutrition knowledge (10.21 ± 1.78 vs. 9.26 ± 2.05, *p* = 0.23) between supplement users and non-users.

## 4 Discussion

This is the first study to perform the assessment and evaluation of sports nutrition knowledge, weight loss strategies, and supplement use in competition climbers at collegiate, national, and international level.

### 4.1 Nutrition knowledge

#### 4.1.1 Knowledge scores

Based on a mean total score of 53.5%, the overall nutrition knowledge of climbers in this study is considered “average,” classified using the scoring system set by Trakman et al. (41). Although no previous data exist in climbing populations, this score is similar, albeit slightly higher than those reported in other sporting populations using the same assessment tool (A-SNKQ), which includes Gaelic games players [46 ± 11.8%, (42)], and a mixed group of Australian football and netball players [47 ± 12%, (36)]. In line with these studies (36, 42), climbing athletes also performed better on the GNK than SNK questions. It is worth noting that there was considerable variation seen in individual scores, particularly in the SNK section with a range of 15–75%, highlighting the need for an individualized approach to athlete support and education.

#### 4.1.2 Individual characteristics and knowledge

##### 4.1.2.1 Sex

Statistical analysis in this sample revealed no significant sex differences for GNK or SNK scores. Previous research assessing sex differences in nutrition knowledge show equivocal results, with some studies suggesting superior knowledge in female athletes (43, 44), whereas other studies, in agreement with the present, show no differences (45, 46).

##### 4.1.2.2 Ability

Although several studies show either no difference in athlete ability level and nutrition knowledge (47, 48), or that higher ability athletes possess greater nutrition knowledge (49, 50), in the present study, post-hoc analysis revealed significantly higher GNK scores in national vs. international level climbers and almost reached significance for the SNK scores. It’s plausible that as lower-level athletes are anticipated to train and travel less than their internationally competitive counterparts, they may be able to commit more time to educational reading or continuation of higher education, however, this was not objectively assessed and should be considered in future research.

##### 4.1.2.3 Age

There was no significant correlation between participant age and GNK, SNK, or combined scores. Previous data in athletes supports this finding (44); however, at least one previous study reported a positive influence of age on GNK scores (36) and the larger age span of the participants in the latter (>36 years), may have helped to reveal a trend in that cohort.

##### 4.1.2.4 Access to dietician

Only ~18% of athletes reported access to a nutritionist/dietitian, with no significant impact of access observed within the combined GNK and SNK scores. This could be interpreted several ways; dietitians may not be effective in communicating nutrition information to these subjects, the subjects may not have utilized dietitian services even though they had access, or the nutrition knowledge survey was not a good proxy for nutrition knowledge that the climbers may have gained from a dietitian’s services. Regardless, since the athletes overall seemed to have average to poor nutrition knowledge, it seems prudent to facilitate access to a dietitian.

#### 4.1.3 Responses to individual items and gaps in knowledge

##### 4.1.3.1 Alcohol

The highest correct response rate was seen within the alcohol category. Not surprisingly, in a population concerned with weight and body composition, almost all climbers recognized alcoholic drinks as a source of “excess” calories that can lead to weight gain. Many also understood the negative implications of alcohol consumption on recovery, which can include reduced muscle protein synthesis and poor sleep quality (51).

##### 4.1.3.2 Macronutrients

Eighty-two percent of climbers in this sample correctly identified that protein needs can be met by a vegetarian diet without the use of supplements. Although this was not assessed in the present study, previous research suggests a relatively high prevalence of vegan or vegetarian athletes within the climbing community (10). Vegan and vegetarian athletes are able to meet all nutrition needs with appropriate planning to achieve good performance outcomes, with no observable effect of protein source (plant-based whole foods + soy protein isolate supplementation vs. mixed whole foods + whey protein supplementation) in supporting muscle strength and mass accrual in response to resistance training, when adequate amounts of protein (≥ 1.6 g/kg/day) are consumed (52).

The majority of climbers in this sample also correctly identified that “Consuming carbohydrate during exercise will assist in maintaining blood glucose levels.” Despite this, current research exploring carbohydrate intake indicates that it is sub-par among adolescent climbers, with 86% of climbing athletes eating below their target carbohydrate intake (8). Similarly, Gibson-Smith (10) reported that adult (>18 years) experienced and elite climbers consumed a daily average intake of 3.7 g/kg of carbohydrate, which is likely inadequate considering the high training volume reported in this study.

##### 4.1.3.3 Micronutrients

Very few athletes correctly answered a question relating to the role of vitamin B in the delivery of oxygen to muscles and were able to correctly identified magnesium and calcium needs, which may have implications for calcium deficiency in higher risk populations, such as vegans (53). Furthermore, there is a positive association between magnesium status and muscle performance, including grip strength, lower-leg power, knee extension torque, ankle extension strength, maximal isometric trunk flexion, rotation, and jumping performance (54); therefore, ensuring magnesium needs are known and met should be a priority for all climbing athletes. Poor micronutrient knowledge has been reported in other athlete groups (36, 55), suggesting this sample of climbers is similar to other athletic populations in terms of their micronutrient nutrition knowledge.

##### 4.1.3.4 Hydration

Athlete knowledge on hydration for sports performance was poor; just 22% of the athletes identified the correct hydration strategy during exercise (i.e., “drink to a plan, based on body weight changes during training sessions performed in a similar climate”), with only 10% of athletes correctly identifying the reason they should drink during exercise (to maintain plasma volume), presenting similar findings to those seen in Australian football and netball athletes (36).

Hydration education could be a method of enhancing climbing performance and preventing adverse events (such as dehydration), which can become apparent with fluid loss as little as 2% of bodyweight during aerobic activity, with impaired repeated anaerobic bouts occurring at ~3% dehydration (56). Interestingly, a recent survey carried in out in climbers (26) reported hydration as one of the “most important” topics, which suggests climbers may feel it is important for performance but unsure how to execute an evidence based hydration strategy.

### 4.2 Weight loss practices

Forty-six percent of males and 38% of female climbers reported intentional weight loss for competition on at least one occasion. Of those who reported weight loss, 75% of females reported doing this 3–5 times, while 17% of males had lost weight more than 10 times. This suggests that losing weight for competitions is not an uncommon practice. Most athletes lost between two to five kilograms over more than two weeks, which based on the mean data obtained, equates to a ~ 3–8% body weight loss.

Approximately 10% of participants reported losing weight ≤ 48 h prior to competition. This is a concerning number, considering that acute weight loss may hinder climbing performance, especially if it is due to dehydration and/or glycogen depletion (56, 57). The weight loss that occurs in about two to four weeks before competition may include some lean muscle, some adipose tissue, and possibly fluctuations in water and glycogen stores. It is reasonable to assume that if lean mass, water, and glycogen are lost, this may hinder climbing performance. In addition, if climbers are in a repeated state of calorie deficit and low energy availability, it puts them at risk for Relative Energy Deficiency in Sport, comprehensively described by the IOC consensus statement (58).

Alarmingly, ~76% (16 of 21) of participants who reported intentionally losing weight for competition are practicing concerning weight loss methods. Skipping meals was the most common form of concerning weight loss practices (~76%), which is similar to what is seen in combat sport athletes (34). Sports professionals working with climbers may need to discuss with the athlete if it is reasonable, safe, and effective to attempt weight loss. Exploring other areas for potential performance gains within training practices, such as strength, flexibility, endurance, climbing technique, sports psychology, fueling and hydration before, during, and after performing, and route reading strategies, may be more efficacious and prudent than weight loss prior to competition.

Notably, the source of influence around weight-loss were from successful athletes, other competitors, and training partners. These sources, unless adequately qualified, are not considered appropriate to safely navigate a climber through a weight loss phase. This is similar to female physique athletes, where in a recent study 89% of athletes relied on a coach for dieting advice, and 73% relied on another athlete (59). It is interesting to note that many accomplished climbers have spoken out regarding their own anecdotal experiences with disordered eating, which is now supported by the scientific literature. For example, (23) reported 43% of elite female climbers scored as “high risk” for disordered eating, with more recent research suggesting the odds ratio for having an injury may be doubled for those with an eating disorder (60). Aspiring athletes emulating elite climbers may find that they are inadvertently emulating an eating disorder, or disordered eating practices. Some climbers reported losing weight using more extreme concerning practices, such as laxative use, restricting fluids, and vomiting, all of which may lead to performance detriment and carry a high risk for short- and long-term adverse health effects relating to dehydration, malnutrition, and injury.

In addition, the belief that low body weight in climbers already at an appropriate weight will lead to better climbing performance is not supported in the literature. A growing number of studies have failed to observe a correlation between weight, BMI, or body fat levels, and climbing ability (8, 10, 61–64). Conversely, in at least one study in female climbers (65), significantly lower body fat percentage values were reported in the elite group, compared to the lower-advanced ability group (23.3% vs. 29.2%, respectively, d = 0.94). However, the authors noted that these values were substantially higher than those reported in elite female competition athletes almost two decades ago [(66); semi-finalists 10.7 ± 1.7% and finalists 9.6 ± 1.9%,] despite performing at the same level. This may reflect a shift in the physiological requirements as the sport has evolved, due to the inclusion of more dynamic and strength reliant moves in the setting of competition routes and therefore, demands a greater focus on absolute power development rather than strength-to-weight ratio alone.

This suggests that the widespread notion that weight loss will lead to better climbing performance may need to be re-examined. There are currently no trials examining whether a climber intentionally losing weight results in performance changes, either through acute manipulation of body weight (i.e., fluid loss, low residue diet) or body weight loss from fat or lean muscle stores. It is important to note that the anthropometric profile of climbers at all levels within the current literature is relatively homogeneous; therefore, our current understanding of the influence of body composition on climbing performance is limited and likely does not apply to individuals who have body fat levels that exceed the parameters of “athletic” norms (67).

### 4.3 Supplement use

Athletes ranked their coach, successful athletes, and internet articles as their top three sources of influence on supplement use. As supplement knowledge appears not to be derived from professional sources, there is higher potential for knowledge to be inaccurate and possibly lead to accidental doping (68). Access to and utilizing dietitians and sports nutrition professionals may be useful for competition athletes to ensure they are taking supplements appropriately, with efficacious dosing and timing strategies, as well as taking clean supplements that comply with WADA and their climbing governing body’s rules. Obtaining supplement knowledge from other sources may be detrimental or influenced by a conflict of interest, particularly when coaches or athletes are involved with sponsorships. Financial gain and sponsorship has been reported as one of the reasons to take supplements among athletes (15).

In previous research among experienced, non-competitive climbers, Gibson-Smith et al. (10) reported that 45% of participants used one or more supplements, with a higher prevalence of use in intermediate/advanced level climbers (57.9%) compared to elite/higher-elite level (38.1%). The most common supplements reported in this 2020 study were protein powder, vitamin D, multivitamins, and fish oil; closely aligning with the most popular supplements used by the present cohort. In the present study, prevalence of supplement use was higher than previously reported (65% vs. 45%) and may be due to the increased demands of training and competition, and the increasing normalization of supplement use in elite sport, where the prevalence is reported to be 81–100% (69).

The three most popular supplements used for performance (creatine, CHO drinks, and beta-alanine) have moderate to strong supporting evidence as ergogenic aids (15) and despite a lack of supplement intervention trials in climbers, are reasonable choices for this sport due to their ability to augment anaerobic capacity, which has been suggested as a determinant of climbing performance (8, 11). No significant differences in supplement use between sexes or competition levels suggest that supplement use is not reserved for only certain tiers of competitive level, but rather many athletes across the board are using them.

Eighty-eight percent of international athletes had access to anti-doping information, which is a reasonable indication that they are aware of the risks of doping. However, access was limited in national and collegiate level climbers and may reflect the scarcity of athlete testing in lower levels of competition. Nevertheless, better access to anti-doping information may be indicated for these athletes. The omission of supplement ingredients, contamination with banned substances, and inaccuracies in supplement content has been well documented, and poses a significant risk for inadvertent doping in athletes (68). Reassuringly, most athletes knew that “supplement labels may contain false or misleading information” (84%).

## 5 Limitations

These self-reported data on anthropometrics, weight loss strategies, and supplements rely on the accuracy of the athletes’ memory, accuracy and clarity of the survey questions, and willingness of the athlete to disclose honest information. As with all self-reported data, there may be inaccurate responses and unreliability, both intentional and unintentional (70). The sensitive nature of some questions (i.e., extreme weight loss methods, admitting to supplement use) may also have inhibited some participants from responding, or answering truthfully (71). Furthermore, despite the development of specific tools, the current status of nutrition knowledge in athletes remains difficult to ascertain (20).

The weight loss survey did not ask for weight loss data other than between <48 h before competition through to >1 month. If an athlete is losing a significant amount of weight over the course of 6–12 months, implementing a well-designed periodized nutrition plan (72), and incorporating strategies to promote the preservation of lean mass, this will likely lead to different performance outcomes than an athlete that loses the same amount of weight over the course of 1 month. Furthermore, the weight loss survey did not ask if the participant had ever had an eating disorder. Although the inclusion criteria did include those “in good health with *no chronic illness that may influence eating patterns*,” which should exclude those with an active, diagnosed eating disorder, but possibly includes those with disordered eating (no formal diagnosis) or a history of eating disorders/disordered eating. Future studies should consider a more explicit description to avoid doubt.

Finally, while the sample obtained allows an insight into the areas examined, the findings should be considered preliminary and not yet generalized to all climbing athletes until further studies and a larger sample of climbers is captured.

## 6 Conclusions and recommendations

These findings suggest that the nutritional knowledge of senior competition climbers is “average”; however, significant gaps in knowledge exist, which include topics such as hydration and micronutrients. Due to the established importance of athlete health and performance, educational programs and/or access to educational resources, should seek to address this. While there appears to be no significant effect of sex or age on knowledge scores, large individual variation is demonstrated, further emphasizing the need for individualized educational and practical nutrition support when working with climbing athletes.

Moreover, this study demonstrated that intentional weight loss for climbing competition is common, with most athletes achieving a ~ 3–8% body weight loss. Alarmingly, a large proportion of athletes who lose weight intentionally for competition report utilizing concerning practices that could increase the risk of both acute and chronic health issues. Furthermore, the most common sources of influence around weight-loss were from inappropriate athlete peers, reflecting the lack of access to professional dietitians. It is crucial that coaches and other professionals working with competitive climbers are vigilant in identifying athletes at risk concerning weight management and establish referral pathways to the appropriate specialist services. Nevertheless, this warrants further investigation.

There is currently no data on body weight manipulation in climbers, and no observable relationship in the current literature between body composition and climbing performance. Thus, athletic individuals should explore other elements, such as physical and physiological capacity (e.g., strength, endurance, flexibility), psychological and psychomotor capability (e.g., route reading, sports psychology, technique), and nutritional intake and timing, to promote long term performance development.

Finally, this data suggests that supplement use is higher in competitive vs. non-competitive athletes assessed in previous research; however, the prevalence of use remains below other elite sports. High quality intervention trials to assess the efficacy of ergogenic aids in climbing remains inadequate, although the current choices of athletes can be justified by their established relevant metabolic effects. Future supplement intervention studies should ensure testing protocols are assessed for reliability and reflect valid determinants of climbing performance. A very large proportion of non-users reported “lack of knowledge” around supplements as a reason, further highlighting the lack of access to credible and reliable sources of information.

## Data availability statement

The raw data supporting the conclusions of this article will be made available by the authors, without undue reservation.

## Ethics statement

The studies involving humans were approved by Sheffield Hallam University Research Ethics Committee (ER10121205). The studies were conducted in accordance with the local legislation and institutional requirements. The participants provided their written informed consent to participate in this study.

## Author contributions

EG-S: Conceptualization, Data curation, Formal analysis, Investigation, Methodology, Writing – original draft, Writing – review & editing. RS: Formal analysis, Writing – review & editing. MM: Writing – review & editing. MR: Supervision, Writing – review & editing.
