# Corrigendum: Nutrition knowledge, weight loss practices, and supplement use in senior competition climbers

**DOI:** 10.3389/fnut.2024.1452189

**Published:** 2024-07-12

**Authors:** Edward Gibson-Smith, Ryan Storey, Marisa Michael, Mayur Ranchordas

**Affiliations:** ^1^Academy of Sport and Physical Activity, College of Health, Wellbeing and Lifestyle, Sheffield Hallam University, Sheffield, United Kingdom; ^2^Sport Industry Research Centre, College of Health, Wellbeing and Lifestyle, Sheffield Hallam University, Sheffield, United Kingdom; ^3^Real Nutrition LLC, West Linn, OR, United States

**Keywords:** climbing, nutrition, bouldering, sport climbing, weight loss, competition, supplements

In the published article, there was an error in the order in which the figure images/graphs are presented as published. Figures 1, 2, 3, 4, and 5 display the incorrect image/graph. The corrected image/graph and its name/caption appear below.

The authors apologize for this error and state that this does not change the scientific conclusions of the article in any way. The original article has been updated.

**Figure 1:**
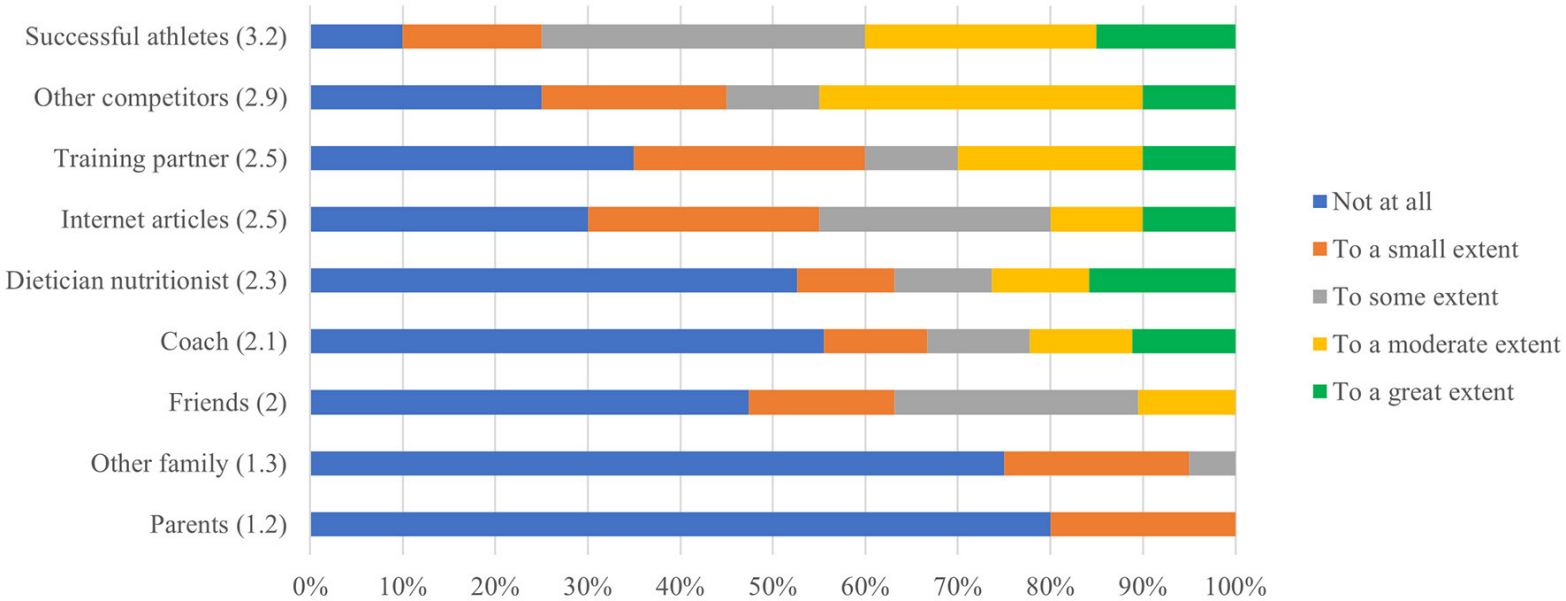
Ranking of influence on weight loss practices.

**Figure 2:**
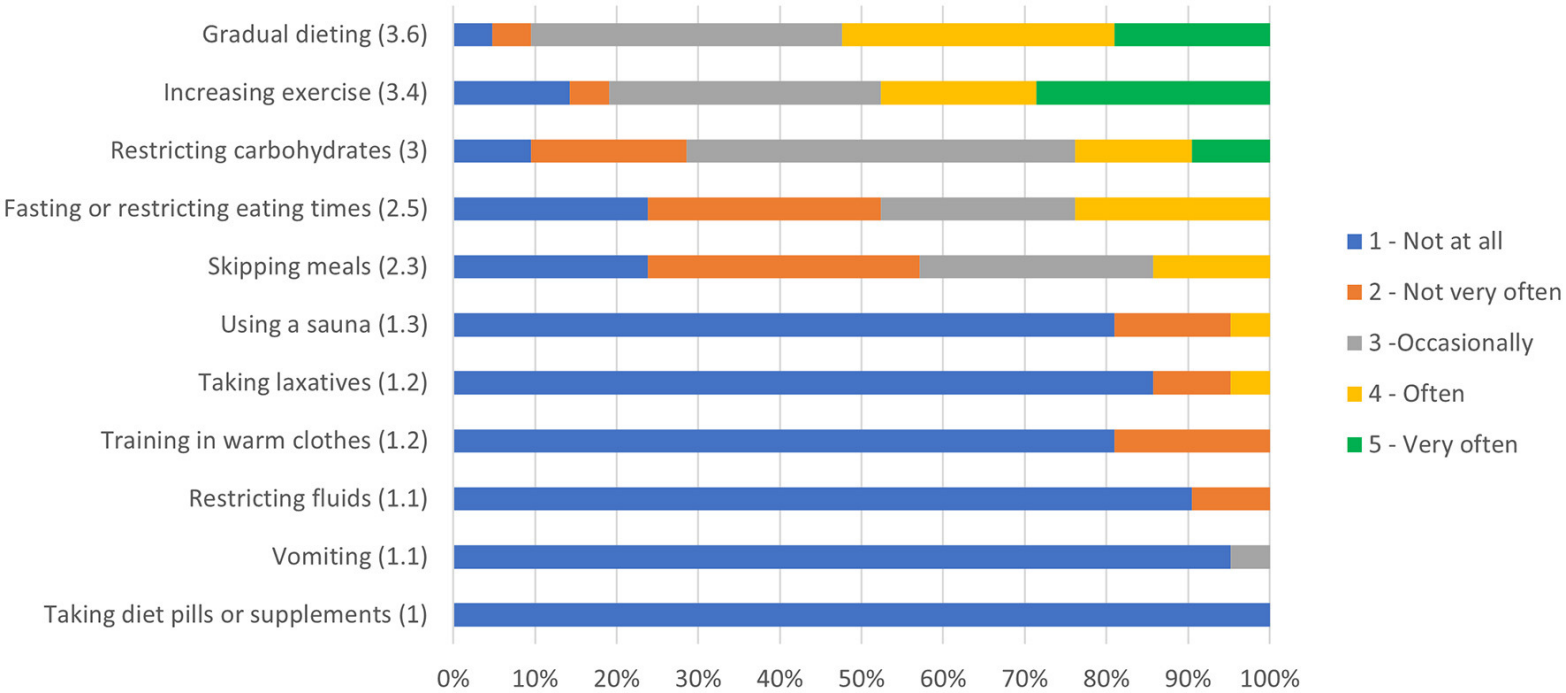
Ranking of prevalence of weight loss practices.

**Figure 3:**
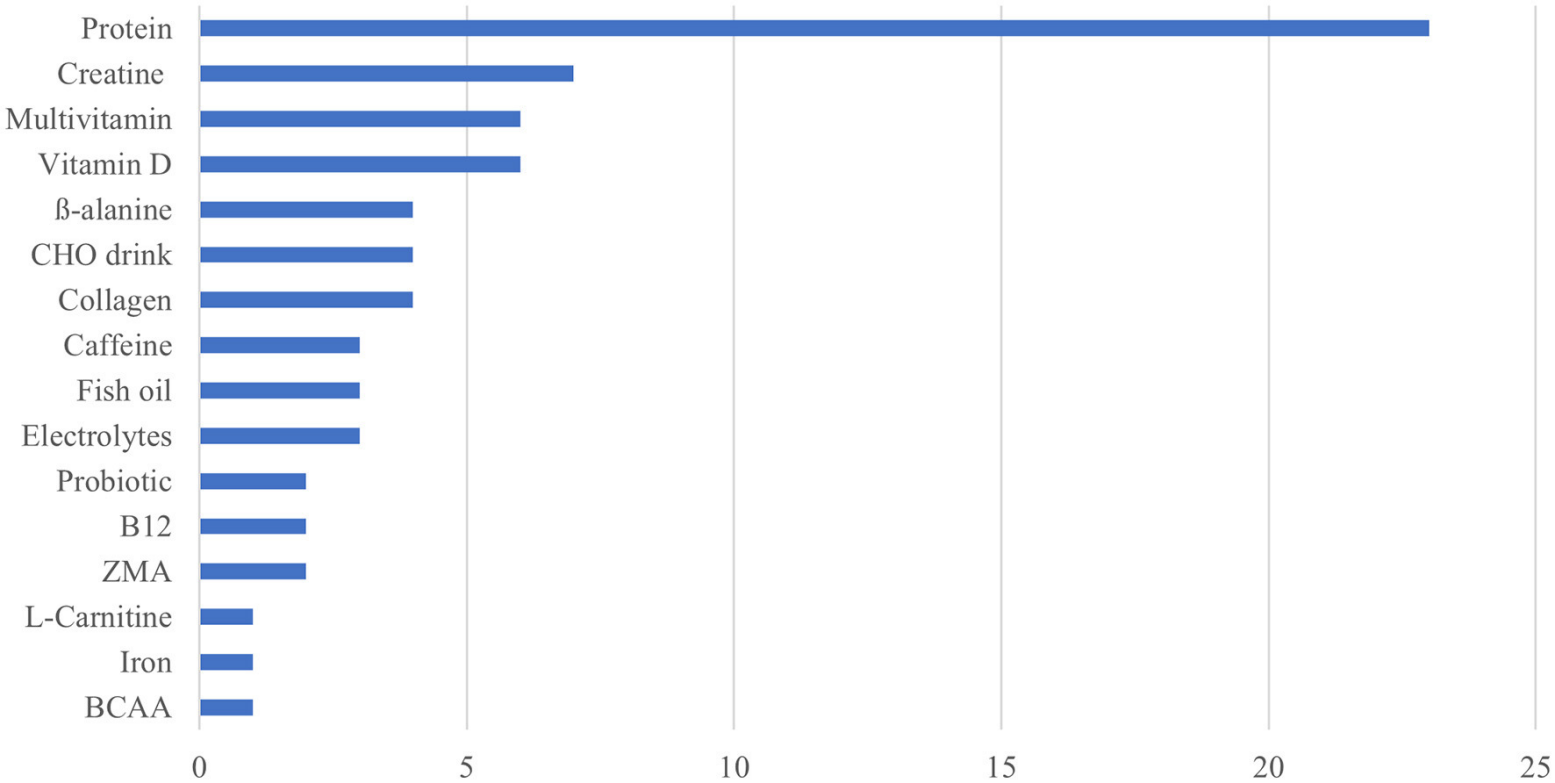
Frequency distribution for the type of nutritional supplements used.

**Figure 4:**
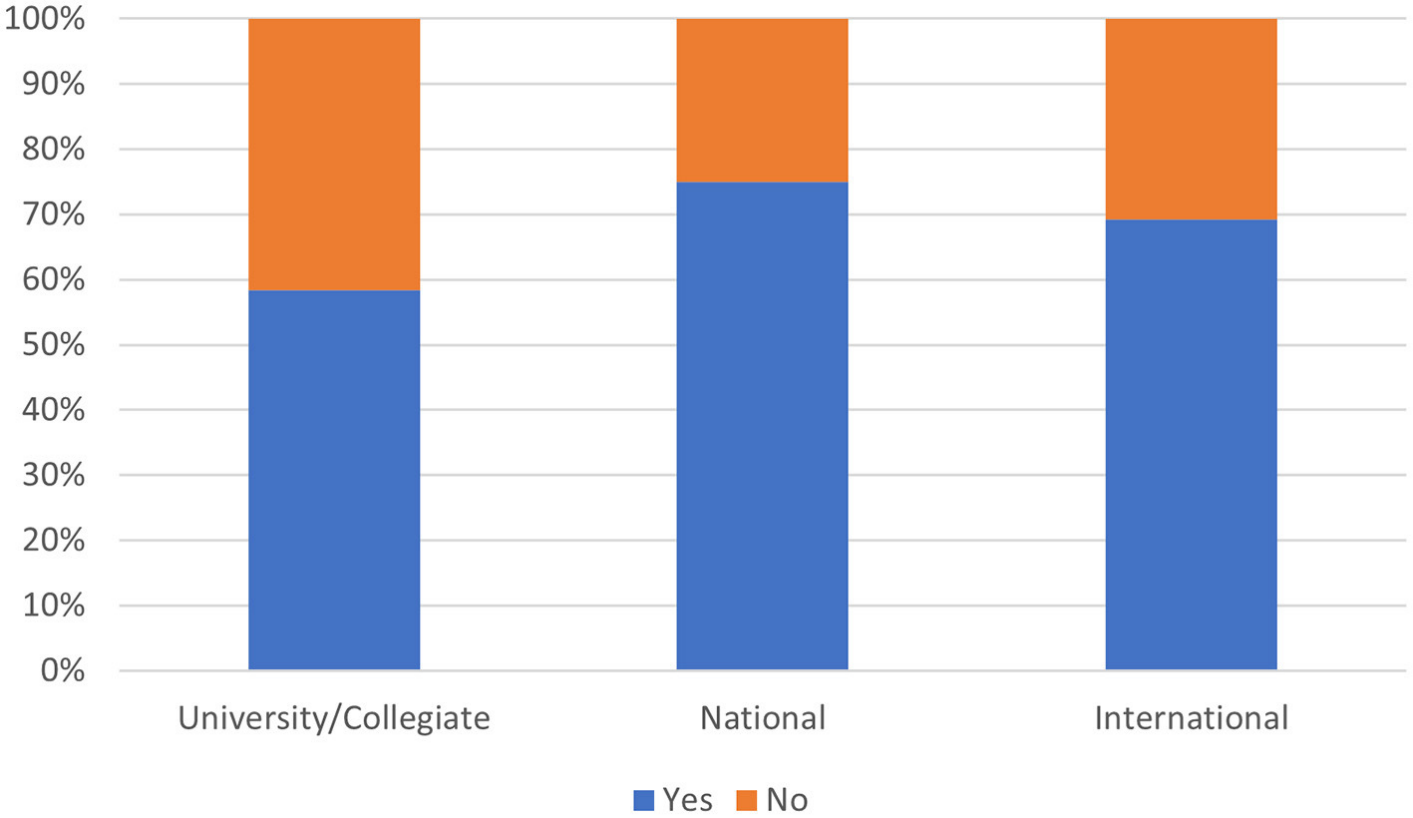
Nutritional supplement use within the competition levels in this sample.

**Figure 5:**
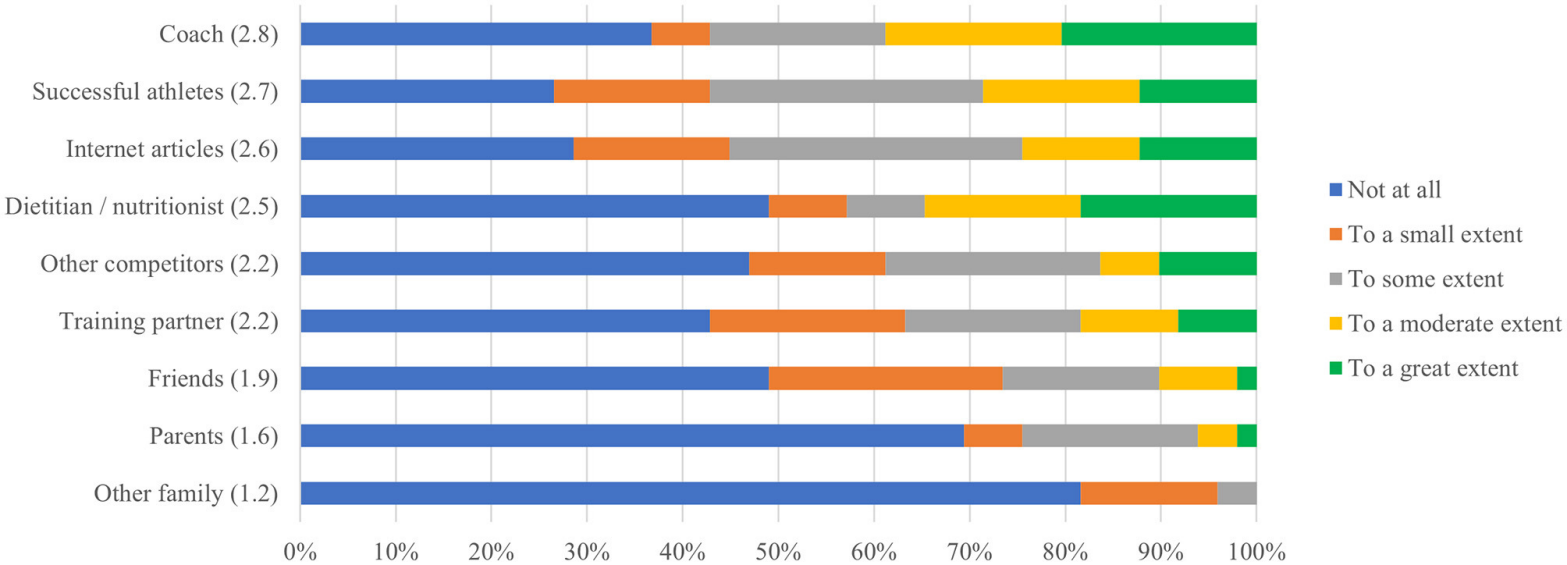
Ranking of influence of supplement use.

