# Supplementary material for: Nutrition knowledge, weight loss practices, and supplement use in senior competition climbers
Source: Front Nutr. 2024 Jan 17;10:1277623. doi: 10.3389/fnut.2023.1277623 (PMC10827858; doi:10.3389/fnut.2023.1277623)
Supplement: Supplementary file 1 [file Data_Sheet_1.PDF]

Sheffield Hallam University | Academy of Sport and Physical Activity

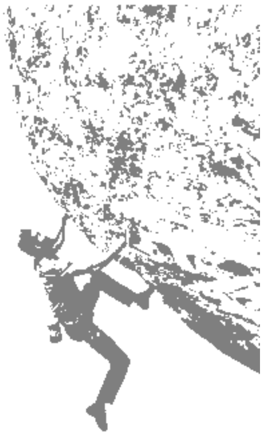

Investigating the Current Sports Nutrition Knowledge, Weight-Loss Strategies & Supplement Use in Competition Climbers

1. Age:

2. Gender: Male ☐ Female ☐

3. What is your nationality?

4. At what age did you begin to climb?

5. At what age did you begin to compete in climbing?

6. How much do you weigh currently?

7. How tall are you?

8. What is your primary climbing discipline?
(the one you currently compete in at the highest level and/or most frequently)

☐ Lead

☐ Bouldering

☐ Speed

Please answer all future questions in this survey in reference to your primary climbing discipline

9. Please describe what level of competition you have competed at.

Local/Regional

☐ Participated ☐ Never participated

National Level

☐ Participated ☐ Never participated

International Level

☐ Participated ☐ Never participated

10. How many times have you competed over the past 12 months?

11. What has been your highest competition finishing place over the past 12 months?

Part 1: Weight Management

1. How much do you weigh **without** a competition coming up? (E.g off-season)

2. Have you lost weight for a competition or competition season?

☐ Yes - Please continue to question 13

☐ No - Please move on to the 'General Nutrition Knowledge' section (Page 3)

3. How many times have you attempted to lose weight over the past 2 years?

4. How much weight would you usually lose for a competition or competition season?

5. What is the most amount of weight you have lost for a competition or competition season?

6. How many days do you typically lose this weight over?

7. At what age did you begin losing weight for competitions?

8. How much weight do you typically lose in the 2 weeks before the competition?

9. Do you attempt to lose weight the day before or on the day of the competition?

10. Using the scale below please rate the amount of influence each source has on your weight management practices

1 = No influence → 5 = High influence

( ) Training partner	( ) Coach	( ) Other competitors
( ) Parents	( ) Dietician/nutritionist	( ) Friends
( ) Internet articles	( ) Successful athletes	( ) Familly

21. The table below presents several methods of weight loss. Using the table below, how often do you use each of the following methods to control or reduce your weight? Tick the box that best describes the frequency. **Your answers will be kept fully confidential.**

Method	Regularly	Sometimes	Almost Never	Never Used
Gradual dieting/restricting calories (≥ 2 weeks)				
Skipping meals				
Fasting or restricting eating times				
Restricting fluids				
Restricting carbohydrates				
Increasing exercise/training				
Using a sauna				
Training in warm clothes				
Taking laxatives				
Taking 'diet pills'				
Vomiting				

Part 2.1: General Nutrition Knowledge

Please circle your answer

Question		Response
Protein eaten in excess of bodily needs can lead to fat gain		Agree / Disagree / Not Sure
Do you think these foods are high or low in carbohydrate?	A banana	High / Low / Not Sure
	½ cup cooked quinoa	High / Low / Not Sure
Fat is required by the body to make molecules involved in immunity		Agree / Disagree / Not Sure
Do you think these foods are high or low in fat?	Cottage cheese	High / Low / Not Sure
	Margarine	High / Low / Not Sure
	Honey	High / Low / Not Sure
Protein absorption in a single sitting is limited		Agree / Disagree / Not Sure
Do you think these foods are high or low in protein?	Yellow Cheese (e.g. cheddar)	High / Low / Not Sure
	Baked Beans	High / Low / Not Sure
	Cooked Quinoa	High / Low / Not Sure
Eggs contain all the essential amino acids needed by the body		Agree / Disagree / Not Sure
Vitamin B is required for efficient delivery of oxygen to muscles		Agree / Disagree / Not Sure
Vitamins provide the body with energy (calories)		Agree / Disagree / Not Sure
When consumed as part of the diet, alcohol contains calories and can lead to weight gain		Agree / Disagree / Not Sure
Drinking large amounts of alcohol can reduce recovery from injury		Agree / Disagree / Not Sure

Part 2.2: Sports Nutrition Knowledge

Please circle your answer

Question	Response
Increasing protein in the diet is the main dietary change needed when only muscle gain is desired	Agree / Disagree / Not Sure
Which do you think is the best lunch option for an athlete trying to gain weight (muscle)?	- A 'mass gainer' protein shake and 3 eggs<br>- Pasta with lean mincemeat and vegetable sauce, plus a dessert of fruit, yoghurt and nuts<br>- A large piece of grilled chicken with salad<br>- A large steak and fried eggs<br>- Not sure
When exercising at low intensities, fat provides almost all the substrate needed to cover energy costs	Agree/Disagree/Not Sure
Vegetarian athletes can meet their protein requirements without the use of supplements	Agree/Disagree/Not Sure
The protein needs of a 100 kg well trained resistance athlete is closest to:	75 g per day / 130 g per day / 250 g per day / They should eat as much protein as possible / Not sure
Athletes have increased magnesium needs due to losses in sweat	Agree / Disagree / Not Sure
The optimal calcium intake for athletes aged 15 to 24 years is 500 mg	Agree / Disagree / Not Sure
A physically fit person eating a nutritionally adequate diet can improve their performance by eating more vitamins and minerals	Agree / Disagree / Not Sure
Vitamin C should be routinely supplemented by athletes	Agree / Disagree / Not Sure
Athletes should drink water during activity in order to:	Maintain sweat volume / Prevent dry mouth / Maintain plasma (blood) volume / All of the above / Not Sure
Regarding fluid intake during physical activity, current recommendations encourage athletes to:	- Drink 50 -100 ml every 15 - 20 minutes<br>- Drink to a plan based on body weight changes during training sessions performed<br>- Drink sports drinks (e.g. Powerade) instead of water when exercising<br>- Suck on ice cubes rather than drinking<br>- Not Sure
Before competition, athletes should aim to consume foods that are high in:	Fluids, fat and carbohydrate / Fluids, fibre and carbohydrate / Fluids and carbohydrate / Not Sure
In events last 60 - 90 minutes, 30 - 60 g of carbohydrates should be consumed per hour	Agree/Disagree/Not Sure
Consuming carbohydrate during exercise will assist in maintaining blood glucose levels	Agree/Disagree/Not Sure
Which of the following best meets the recommendations for a snack consumed during	A protein shake / A ripe banana / 2 Boiled eggs / A handful of nuts / Not Sure

high-intensity exercise lasting around 90 minutes	
How much protein do you think experts recommend athletes should have AFTER completing a resistance exercise session?	- 1.5g/kg body weight (150 – 130 g)<br>- 1.0 g/kg body weight (50 - 100 g)<br>- 0.3g/kg body weight (15 - 25 g)<br>- Not Sure
Supplement labels may contain false or misleading information	Agree / Disagree / Not Sure
The purity and safety of all supplements are tested before sale	Agree / Disagree / Not Sure
In relation to improving sporting performance, which of the following supplements do you think has NOT been supported by a strong body of scientific evidence?	Caffeine / Ferulic acid / Bicarbonate / Creatine / Not Sure
Which of the following supplements do you think is banned?	Caffeine / Bicarbonate / Carnitine / Testosterone / Not Sure

Part 3: Supplement Use

The following section asks about your use of nutritional supplements in the last 6 months. The term 'supplements' refers to any product intended to supplement the diet, provide nutrients and/or enhance performance such as vitamins, minerals, carbohydrate sports drinks/ bars, amino acids, herbal remedies, creatine and caffeine etc.

1. Have you used any nutritional supplements in the last 6 months?

Yes ☐ Please go to question 2

No ☐ Please go to question 4

2. For each supplement that you have used in the last 6 months please provide the following information:

- **Identify whether you take the supplement for <u>HEALTH</u> or <u>PERFORMANCE</u>**

- **Supplement type?** e.g sports drinks, gels/bars, creatine, protein, vitamins, minerals, probiotics etc

- **How often do you take the supplement?** e.g daily, weekly

- **When do you take the supplement?** e.g training / rest days / competition

- **Timing?** e.g before, during, or after a training or competition/ before bed / in the morning

- **How much?** Do you know the dose / how many grams/ scoops / pills a day?

- **Reason for taking the supplement?** e.g medical requirement / to support immune system / to provide energy / increase strength or power / to aid recovery / because everyone else does / you are told to

Examples:

Performance: "I drink 1 x sports drink (Lucozade sport 500 ml) during my training session if lasting more than 60 mins to provide energy"

Health: "I take 1 x Probiotic capsule (Healthspan Super50) every morning to support my immune system"

Performance: "I take 5 grams of creatine monohydrate (Myprotein) every morning to improve my strength and power

………

………

………

………

………

………

………

………

………

………

………

………

3. Where did you obtain / buy your supplements? **(Please check all that apply)**

Provided by a team sponsor	☐	Provided by a sports nutritionist / dietitian	☐
From a supermarket	☐	From a health food/sports shop	☐
From a pharmacy	☐	I ordered them on the internet	☐
Other (Please state below)	☐		

…………………………………………………………………………………………………

4. If you don't use supplements, why not? **(Please check all that apply)**

I do not need them	☐	They are unhealthy	☐
I don't know enough about them	☐	I am concerned about drugs testing	☐
They are too expensive	☐	My sport does not allow them	☐
Taking supplements is like cheating	☐	Other (Please state below)	☐

……….

5. Have you **EVER** experienced any negative/side-effects from using a supplement? E.g reduced performance, rapid bowel movements, feeling sick, cramps etc

Yes ☐ Continue to question 6

No ☐ Please continue to Question 7

6. Which product(s) did you use and what were the negative/side effects?

………

………

………

………

………

7. Do you have access to information on anti-doping?

Yes ☐

No ☐

8. Do you have access to a sports nutritionist / dietitian through your sport or team?

Yes ☐

No ☐

9. Using the scale below please rate the amount of influence <u>each source</u> has on your supplement use.

1 = No influence → 5 = High influence

( ) Training partner ( ) Coach ( ) Other competitors

( ) Parents ( ) Dietician/nutritionist ( ) Friends

( ) Internet articles ( ) Successful athletes ( ) Family

Please indicate to what extent you agree or disagree with the following statements:

10. ‘The more supplements I take, the better I will perform’.

Strongly disagree ☐

Disagree ☐

Neither agree nor disagree ☐

Agree ☐

Strongly agree ☐

11. ‘Taking supplements gives me the competitive edge I need to win’.

Strongly disagree ☐

Disagree ☐

Neither agree nor disagree ☐

Agree ☐

Strongly agree ☐

12. ‘Exercise increases the need for supplements’.

Strongly disagree ☐

Disagree ☐

Neither agree nor disagree ☐

Agree ☐

Strongly agree ☐

13. ‘There is a risk of consuming a banned substance when taking a supplement’.

Strongly disagree ☐

Disagree ☐

Neither agree nor disagree ☐

Agree ☐

Strongly agree ☐

14. ‘I feel pressured to take nutritional supplements because my competitors/ opponents do’

Strongly disagree ☐

Disagree ☐

Neither agree nor disagree ☐

Agree ☐

Strongly agree ☐

Many thanks for taking part in this research and contributing to the development of your sport.

Please enclose and seal your completed questionnaire & signed consent form in the envelope provided and hand back to the principal investigator.
